# Construction of an anaplastic thyroid cancer stratification signature to guide immune therapy selection and validation of the pivotal gene HLF through *in vitro* experiments

**DOI:** 10.3389/fimmu.2024.1478904

**Published:** 2025-01-13

**Authors:** Li Pengping, Yin Kexin, Xie Yuwei, Sun Ke, Li Rongguo, Wang Zhenyu, Jin Haigang, Wang Shaowen, Huang Yuqing

**Affiliations:** ^1^ Department of Thyroid & Breast Surgery, The First People’s Hospital of Xiaoshan District, Xiaoshan Affiliated Hospital of Wenzhou Medical University, Hangzhou, Zhejiang, China; ^2^ The First Affiliated Hospital of Anhui Medical University, Hefei, Anhui, China; ^3^ Neuromedicine Center, The University of Hong Kong-Shenzhen Hospital, Shenzhen, Guangdong, China

**Keywords:** anaplastic thyroid cancer (ATC), T cell immunity, machine learning, prediction, model

## Abstract

**Introduction:**

While most thyroid cancer patients have a favorable prognosis, anaplastic thyroid carcinoma (ATC) remains a particularly aggressive form with a median survival time of just five months. Conventional therapies offer limited benefits for this type of thyroid cancer. Our study aims to identify ATC patients who might bene t from immunotherapy.

**Methods:**

Our study uses multiple algorithms by R4.2.0, and gene expression and clinical data are collected from TCGA, GEO and local cohort. In vitro experiments, such as western blot and immunofluorescence staining, are performed.

**Results:**

Using a set of five genes uniquely expressed across various types of thyroid cancer, we developed a machine-learning model to distinguish each type within the GEO dataset of thyroid cancer patients (GSE60542, GSE76039, GSE33630, GSE53157, GSE65144, GSE29265, GSE82208, GSE27155, GSE58545, GSE54958, and GSE32662). These genes allowed us to stratify ATC into three distinct groups, each exhibiting significantly different responses to anti-PD1 therapy as determined by consensus clustering. Through weighted gene co-expression network analysis (WGCNA), we identified 12 differentially expressed genes closely associated with immunotherapy outcomes. This led to the creation of a refined signature for predicting ATC’s immune responsiveness to anti-PD1 therapy, which was further validated using thyroid cancer cohorts from TCGA and nine melanoma cohorts from clinical trials. Among the 12 genes, HLF stood out due to its strong association with various cancer hallmarks.

**Discussion:**

Our study revealed that HLF impedes ATC progression by down-regulating the epithelial-to-mesenchymal transition (EMT) pathway, reducing T cell exhaustion, and increasing sensitivity to sorafenib, as demonstrated through our *in-vitro* experiments.

## 1 Introduction

Although the prognosis for most thyroid cancer patients is favorable, anaplastic thyroid carcinoma (ATC) remains a notably aggressive form with a median survival time of only five months (1, 2). ATC comprises only 2% of all types of thyroid cancers but accounts for a disproportionate 14% to 39% of deaths associated with this cancer (3). For patients who outlive the median survival time, conventional therapies such as chemotherapy and radiotherapy offer limited benefits (4, 5). Besides, early diagnosis of ATC presents another significant challenge. Thus, detecting ATC in its initial stages and finding other effective therapies are both crucial for improving patient outcomes.

It has been reported that resistance to chemotherapy and radiotherapy is the main reason for poor survival. For some patients carrying the BRAF and MEK gene mutation, the combination of dabrafenib and trametinib has shown promise in extending the effective treatment period (6). For those patients without these mutations, immunotherapy (anti-PD-1 and anti-PD-L1) has manifested a 1-year survival rate of approximately 40%, making it the most promising known therapy (7). However, more research is still urgently needed to identify the subgroup of patients who can benefit from the immunotherapy.

Our study endeavors to identify ATC patients who could potentially benefit from immunotherapy. Leveraging a set of five uniquely expressed genes across various types of thyroid cancer, our research has developed a machine-learning model capable of distinguishing each type within the GEO dataset of thyroid cancer patients (GSE60542, GSE76039, GSE33630, GSE53157, GSE65144, GSE29265, GSE82208, GSE27155, GSE58545, GSE54958, and GSE32662). Utilizing these genes, we have stratified ATC into three distinct groups, each demonstrating significantly different responses to anti-PD1 therapy. Additionally, we employed weighted gene co-expression network analysis (WGCNA) to identify 12 differentially expressed genes intimately associated with both the grouping and immunotherapy outcomes. This led to the creation of a refined signature that could more accurately predict ATC’s immune responsiveness to anti-PD1 therapy, which was further corroborated using thyroid cancer cohorts and 9 melanoma cohorts from the clinical trial. Among the 12 genes analyzed, HLF emerged as significantly associated with various cancer hallmarks. Our study elucidated the mechanism by which HLF impeded anaplastic thyroid carcinoma (ATC) progression. Specifically, HLF down-regulated the epithelial-to-mesenchymal transition (EMT) pathway, reduced T cell exhaustion, and increased sensitivity to sorafenib, as demonstrated by our *in-vitro* experiments.

## 2 Methods

### 2.1 Data collection

Gene expression profiles and clinical characteristics of thyroid cancer were collected from the cancer genome atlas (TCGA, https://portal.gdc.cancer.gov) and gene expression omnibus (GEO, https://www.ncbi.nlm.nih.gov/geo/). A total of 506 samples with follow-up data were collected from TCGA, and 715 samples were collected from GEO datasets GSE27155, GSE58545, GSE76039, GSE82208, GSE60542, GSE29265, GSE33630, GSE65144, GSE53157, GSE54958, and GSE32662. Among these, 114 samples were thyroid non-cancerous tissue (TNC), 78 samples were anaplastic thyroid carcinoma (ATC), 225 samples were papillary thyroid carcinoma (PTC), 40 samples were follicular thyroid carcinoma (FTC), and 54 samples were medullary thyroid carcinoma (MTC). Verified cohorts of clinical trials (anti-PD1) were collected from GSE115821 (N=37), GSE78220 (N=28), GSE91061 (N=109), Nathanson_2017 (N=24), phs000452 (N=153), and PRJEB23709 (N=91).

### 2.2 Bioinformatic analysis

#### 2.2.1 Subgrouping signature construction

GEO cohorts of thyroid cancer (TC) were used to identify differentially expressed genes (DEGs) in ATC using R4.2.0 (package: DESeq2). The ConsensusClusterPlus package in R4.2.0 was employed to perform consensus clustering analysis based on the 5 DEGs (BCL2, BHLHE40, MICAL2, TGM2, TPO) (parameters: maxK=10, reps=50). The consensus cumulative density function (CDF) and delta area indicated that a 3-subgroup division was the optimal outcome.

#### 2.2.2 Subtype identification model construction

Decision curve analysis (DCA) was used to evaluate the value of the 5-DEGs in identifying ATC or other subgroups of TC using the ggDCA package in R4.2.0. The DALEX, randomForest, kernlab, xgboost, caret, and pROC packages were applied to construct identification models.

#### 2.2.3 Tumor immune index calculation

Infiltration immune cell fractions were predicted using CIBERSORT, ssGSEA (single sample GSEA), and Pompimol Charoentong’s algorithm in R4.2.0. The immune score was predicted using the estimate package in R4.0. Tumor Immune Dysfunction and Exclusion (TIDE) and anti-PD1 response were predicted using the online tool (http://tide.dfci.harvard.edu). To assess MeTIL characteristics, the individual methylation values of MeTIL markers were converted to MeTIL scores using principal component analysis (PCA). The data were converted to a unit-free Z-Score by applying the formula (x-μ)/σ. According to the median value of PDCD1, the samples were divided into high and low-expression groups. Wilcoxon Rank Sum Tests were used to compare the MeTIL scores between the two groups (8).

TIP (Tracking Tumor Immunophenotype) was a meta-server that systematically integrates two existing third-party methods, “ssGSEA” and “CIBERSORT”, for tracking, analyzing, and visualizing the anti-cancer immune state and the proportion of immune-infiltrating cells in the seven steps of the cancer immune cycle using RNA-seq or microarray data. Spearman correlation between genes and TIP scores, as well as the autocorrelation between TIP scores, were calculated, and the linkET package was used for visualization (8). The red and green lines represented positive and negative correlations, respectively, while the gray lines indicated no significance. The thickness of the lines represented the absolute value of the correlation coefficient. The correlation in the triangular region was represented by the color depth and size of the square: red/blue indicated a positive/negative correlation, with darker colors signifying more significant P-values, and larger squares representing greater absolute values of the correlation coefficient. Easier was a tool for predicting biomarker-based immunotherapies (Cytolytic score, CYT; Tertiary lymphoid structures, TLS; IFNy, T cell-inflamed, Chemokines) based on cancer-specific immune response models, aiming to predict anti-tumor immune responses from RNA-seq data. The CYT level of TCGA-BLCA was calculated using the Easier package to evaluate CYT characteristics. According to the median value of PDCD1, the samples were divided into high and low-expression groups (9). Wilcoxon Rank Sum Tests were used to compare the CYT scores between the two groups.

#### 2.2.4 Cell signaling score calculation

The CancerSEA database collated 14 different functional states of tumor cells (10). The Z-score algorithm, proposed by Lee et al. (11), integrated characteristic gene expression to reflect the activity of a given pathway. Fourteen functional state gene sets were calculated using the Z-score algorithm in the R-package GSVA. The values of each gene set were enumerated separately as Z-scores. Pearson correlations between genes and the Z-scores of each gene set were then calculated.

#### 2.2.5 Pan-cancer analysis of HLF expression

The expression level of HLF in pan-cancer was calculated by R4.2.0 (TCGA cohorts).

#### 2.2.6 The 12-gene signature construction

Firstly, WGCNA (Weighted Gene Co-Expression Network Analysis) was applied to screen out hub genes using the WGCNA package in R4.2.0. The most correlated gene sets (both negative and positive) were collected for subsequent machine learning. AI modeling for ATC subgroup stratification was developed using six AI functions: extreme gradient boosting (XGboost, xgboost package in R4.2.0), support vector machine (SVM, e1071 packages in R4.2.0), multi-logistic (nnet packages in R4.2.0), random forest (RF, randomForest package in R4.2.0), deep learning (DL, h2o package in R4.2.0), and K-Nearest Neighbor (KNN, kknn package in R4.2.0). During model construction, 75% of the data was randomly selected as the training cohort, and 25% was randomly selected as the testing cohort. Gene expression values were standardized to range from 0 to 1 using the preProcess function (caret and tidyverse packages).

### 2.3 Biological experiments

#### 2.3.1 Clinical sample collection

Twenty-two thyroid cancer samples were collected from 2021-12-01 to 2022-08-01. All experiments were approved by the Medical Ethics Committee of The First People’s Hospital of Xiaoshan District, Xiaoshan Affiliated Hospital of Wenzhou Medical University. All patients with ATC were confirmed by at least two pathologists.

#### 2.3.2 Multiple immune fluorescence staining

The procedures for paraffin embedding, tissue sectioning, and immunohistochemistry for HLF, CD8, and PD1 expression levels were performed as previously described (PMID: 23200678 and 20571492). The working concentrations of antibodies against HLF (Proteintech, Wuhan, China), CD8 (Abcam, Shanghai), and PD1 (Proteintech, Wuhan, China) were 1:150. The protein expression levels were assessed by Mean of Integrated Option Density (IOD) with Image-Pro Plus. Briefly, the area of interest (AOI) was detected to gain the Mean of IOD (IOD/AOI, MI).

#### 2.3.3 Reagents

Sorafenib was purchased from CSNpharm (A316727) and dissolved in PBS. Antibodies against beta-actin (AF7018, Affinity), CD8 (GB15068, Servicebio), HLF (DF7892, Affinity), N-cadherin (AF5239, Affinity), E-cadherin (BF0219, Affinity), Vimentin (BF8008, Affinity), Twist1 (AF4009, Affinity), Snail1 (AF6032, Affinity), PD-L1 (BF8035, Affinity), phosphorylated-JAK3 (p-JAK3, AF8160, Affinity), JAK3 (AF0008, Affinity), p-STAT3 (AF3293, Affinity), and STAT3 (BF6294, Affinity) were used for western blot.

#### 2.3.4 Cell culture

ATC cell lines (CAL62, TCO1) were obtained from the cell bank of the Chinese Academy of Science in 2022 with STR matching analysis. The culture media for both ATC cell lines were DMEM with 10% fetal calf serum and 100 units/mL penicillin and streptomycin.

#### 2.3.5 Small interfering RNA experiments

5 × 10^5 ATC cells were transplanted into 6-well plates for 24 hours and then transfected with three different sequences of HLF siRNA (GenePharma, Shanghai, China) for 48, 72, and 96 hours using Lipofectamine 3000 reagent (Invitrogen, USA) and Opti-MEM (Life Technologies, USA), according to the manufacturer’s instructions for optimal transfection efficiency. The three siRNA sequences for HLF were as follows:

Sequence-1
Forward (5′-3′): TGCAAAATGTTCAAAATTGAA
Reverse (5′-3′): CAATTTTGACATTTTGCTAA

Sequence-2
Forward (5′-3′): ATTAAAAAAAAACTTTTCGGGTC
Reverse (5′-3′): CGAAAGTTTTTTTTTAATAT

Sequence-3
Forward (5′-3′): AAATGTTGCTGAGCTTTTCCT
Reverse (5′-3′): GAAAGCTCAGCAACTTTTA

#### 2.3.6 Western blot

Total protein extraction: Cells were harvested using a cytology brush and lysed with RIPA lysis buffer (Sigma, USA) supplemented with phosphorylase and protease inhibitor mixture (Thermo, USA), and quantified by the BCA assay. Cytoplasmic and nuclear protein extraction: Cells were harvested using trypsin (Invitrogen), then cytoplasmic and nuclear proteins were extracted using the Cytoplasmic and Nuclear Protein Extraction Kit (Thermo Scientific, USA) according to the protocol, and quantified by the BCA assay. Protein samples were separated by SDS-PAGE (EpiZyme, China, PG113) and transferred to a 0.45 μm or 0.20 μm pore-sized PVDF membrane (Millipore, USA). The membranes were blocked with Tris-buffered saline containing 5% skim milk powder (Biosharp, BS102-500 g, China) at room temperature for 1 hour, followed by incubation with primary antibodies at 4°C overnight. The next day, after three washes with TBST solution, the membranes were incubated with secondary antibodies at room temperature for 60 minutes. Finally, immunoreactive bands were detected using an enhanced chemiluminescence kit (Biosharp, BL520B, China). The conjugation yield was calculated via gel band quantification using Image J software (12).

#### 2.3.7 Migration ability assays

For trans-well assays, 50,000 cells, with or without special treatments, were transplanted into trans-well plates (24-well, 8.0μm, Corning Incorporated, Corning, NY, USA) with a 10% gradient of fetal calf serum for 48 hours. After 24 hours of incubation, the cells that had migrated to the lower surface of the filter were fixed with 4% paraformaldehyde and stained with hematoxylin and eosin. The stained cells were then observed and photographed using a light microscope. Quantification of the passed cell area was performed using Image-Pro Plus (13).

#### 2.3.8 Live & dead cell staining

Live and dead cell staining was carried out using Calcein AM/PI staining. After being seeded in a 24-well plate and cultured for 24 hours, ATC cells were treated with DMSO or 5μM GEM for another 48 hours. Then, all cells were co-cultured with Calcein AM and PI and observed at 480 nm and 525 nm, respectively.

#### 2.3.9 PBMCs extraction

Simply, PBMCs were isolated via Ficoll-Paque density gradient centrifugation: 5 mL of peripheral blood was collected from healthy female volunteers, diluted with PBS at a 1:1 ratio, followed by gentle mixing. Add 10 mL of the diluted blood to 2 mL of Ficoll liquid (density 1.077). The clear stratification of blood and Ficoll liquid confirmed success. Carefully transferred the sample to the centrifuge and spin at 500 g for 15 minutes. Removed the centrifuge tube with care, aspirate the white thin film layer in the middle, representing individual nucleated cells. Wash the isolated nucleated cells with 10 mL of PBS, centrifuge at 250 g for 10 minutes, and discarded the supernatant. Repeat the washing step once and the suspended cells were frozen in vials at 100 million cells/mL in HI FBS with 5% DMSO after washing. Stored in liquid nitrogen, they were revived gradually and washed in pre-warmed RPMI with FBS and pen/strep. Following a 4-5 hour incubation at 37°C, viability was assessed using Trypan blue (0.1%).

#### 2.3.10 Flow cytometry

The co-cultured PBMC were stained with Fixable Viability Stain (Thermo, L34965) and Fc receptor blocking reagent [Ultra-LEAF™ Purified anti-mouse CD16/32 (101320, BioLegend)]. Next, they were stained with CD-3 (BD 557943), PD-1 (BD 561273), and CD8 antibody (thermo, A15448). The prepared single-cell suspensions were filtered through 40-μm nylon meshes (352340, Corning). Results were then acquired using BD Calibur, BD Fortessa, or Miltenyi MACSQuant systems. Data were analyzed with FlowJo_V10 software (TreeStar).

### 2.4 Statistical analysis

All data analyses were performed in R4.2.0. Pearson’s test was used to calculate the correlation between different genes. Wilcox rank sum test and Kruskal-Wallis rank sum test were used to assess differences in continuous variables. Univariate Cox regression was performed to calculate the hazard ratio (HR), and the log-rank test was used to compare survival differences. Heatmaps were generated using the pheatmap package in R4.2.0. Receiver operating characteristic (ROC) curves and AUC values were generated using the pROC package in R4.2.0. GO and KEGG analyses were performed using the clusterProfiler package in R4.2.0. P<0.05 was considered to indicate a statistically significant difference.

## 3 Results

### 3.1 Five distinct genes were expressed significantly between any pair of thyroid cancer subtypes

**Figure 1:**
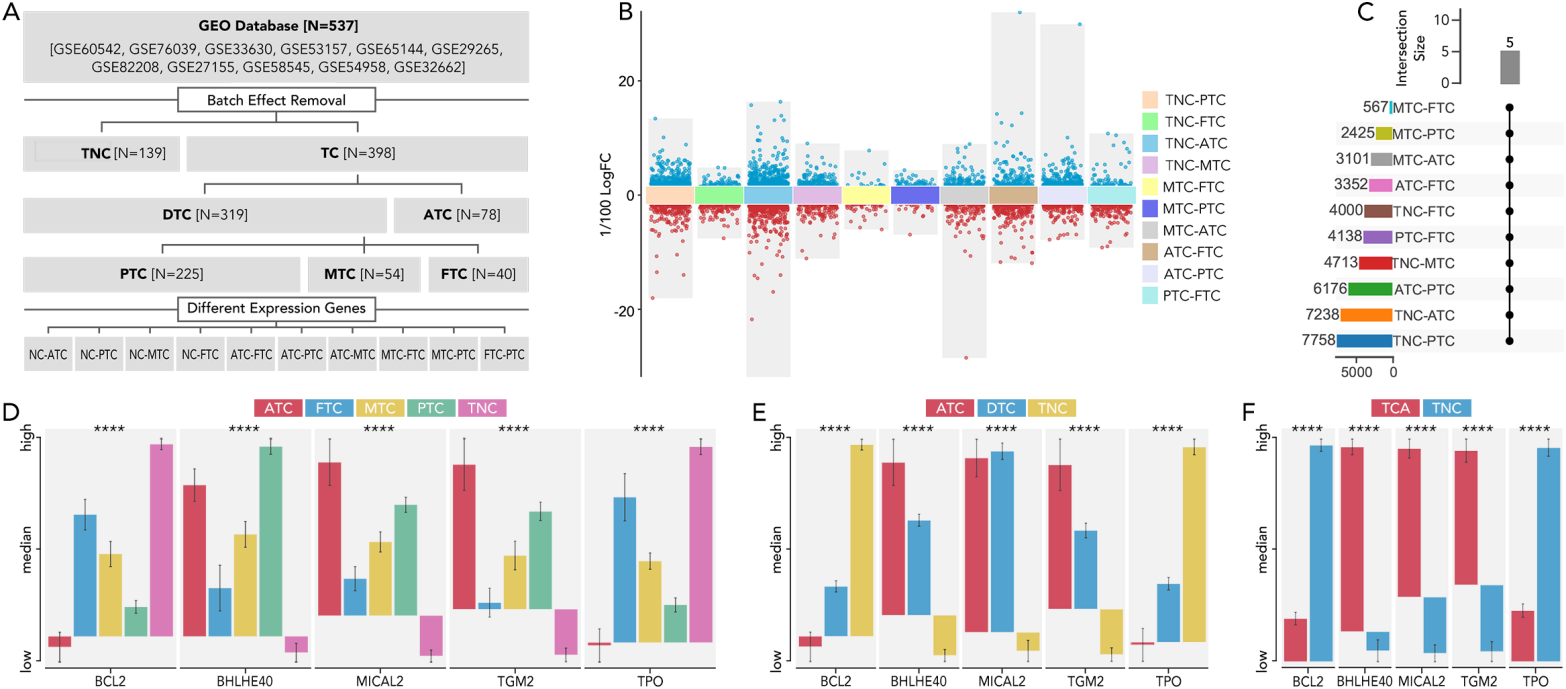
The 5 distinct genes were expressed differently in each type of thyroid cancer. **(A)** Flow chart illustrating the thyroid dataset acquisition and comparison strategies in TCA. **(B)** The analysis of differentially expressed genes was conducted between various thyroid cancer subtypes and NC. The comparisons included TNC vs. PTC, TNC vs. FTC, TNC vs. ATC, TNC vs. MTC, MTC vs. FTC, MTC vs. PTC, MTC vs. ATC, ATC vs. FTC, ATC vs. PTC, and PTC vs. FTC. The criteria for significance were set at a P value > 0.05 and a fold change (|FC|) > 2. **(C)** The number of differentially expressed genes in each comparison was listed, with 5 genes consistently differentially expressed across all comparisons. **(D)** The expression levels of these five genes were compared among ATC, FTC, MTC, PTC, and TNC. **(E)** The expression levels of these five genes were compared among ATC, DTC, and TNC. **(F)** The expression levels of these five genes were compared among ATC, DTC, and TNC thyroid carcinoma (TCA), differentiated thyroid carcinoma (DTC), anaplastic thyroid carcinoma (ATC), papillary thyroid carcinoma (PTC), medullary thyroid carcinoma (MTC), follicular thyroid carcinoma (FTC), thyroid non-cancerous tissues (TNC), fold change (FC) P value < 0.0001 (****).

The main available gene expression data of 537 thyroid cancer samples from GEO database (GSE60542, GSE76039, GSE33630, GSE53157, GSE65144, GSE29265, GSE82208, GSE27155, GSE58545, GSE54958, and GSE32662) were extracted for analysis of differentially expressed genes between different subtypes of thyroid cancer (**Figure 1A**). Multiple comparisons between each group identified five genes (BCL2, BHLHE40, MICAL2, TGM2, TPO) that are significantly differentially expressed (P value > 0.05 and a fold change (|FC|) > 2) in any pair of all the subtypes (thyroid carcinoma (TCA), differentiated thyroid carcinoma (DTC), anaplastic thyroid carcinoma (ATC), papillary thyroid carcinoma (PTC), medullary thyroid carcinoma (MTC), follicular thyroid carcinoma (FTC), thyroid non-cancerous tissues (TNC) (**Figures 1B–F**).

### 3.2 The model based on the 5 genes by machine learning can distinguish each subtype well

**Figure 2:**
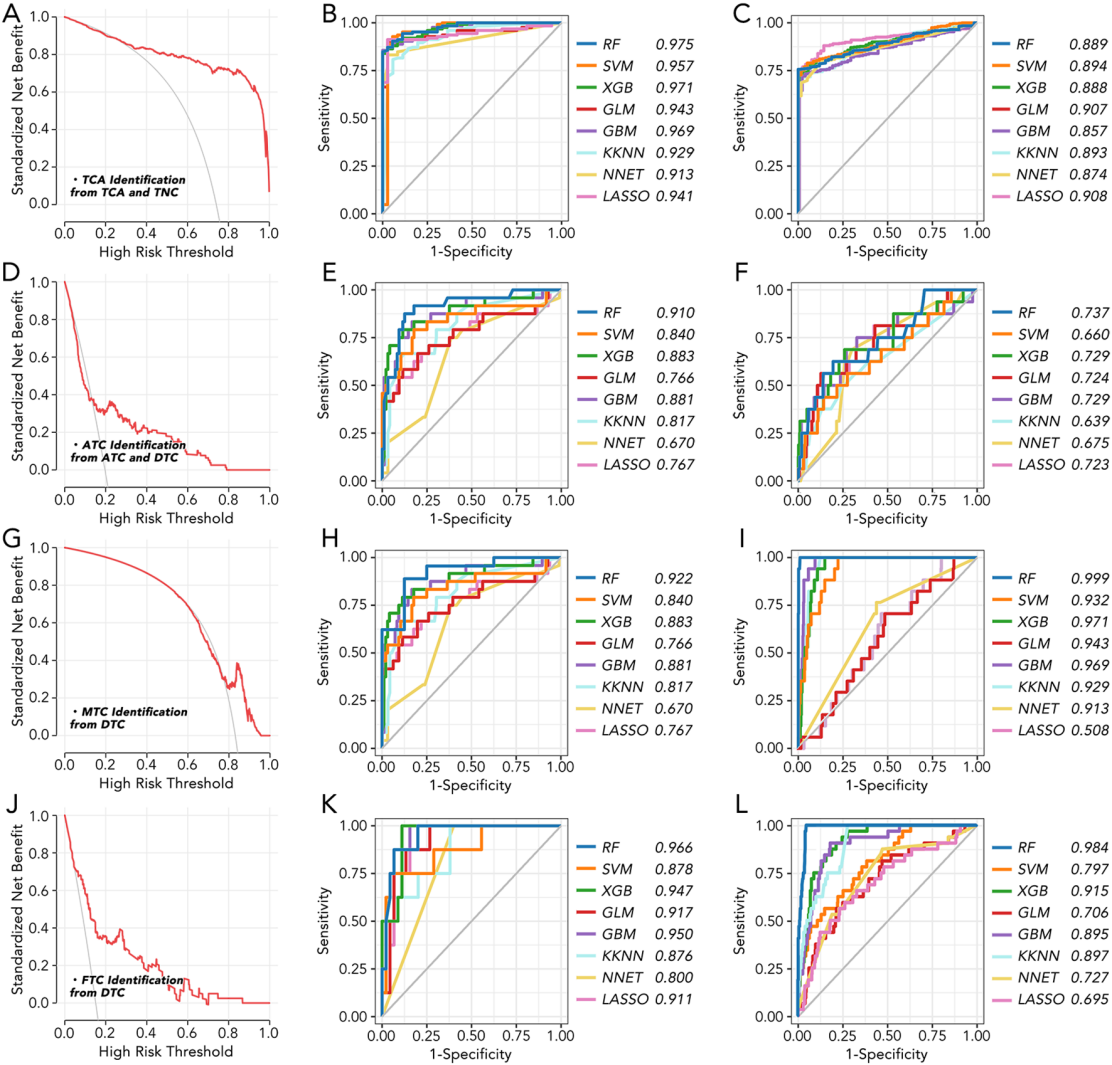
The machine learning model for thyroid cancer classification based on 5 key genes **(A)** The standardized net benefit and high-risk threshold were calculated between TCA and TNC. **(B)** Machine learning model to distinguish TCA from TNC in GEO datasets based on 5 key genes. **(C)** Machine learning model to distinguish TCA from TNC in TCGA based on 5 key genes. **(D)** The standardized net benefit and high-risk threshold were calculated between ATC and DTC. **(E)** Machine learning model to distinguish ATC from DTC in GEO datasets based on 5 key genes. **(F)** Machine learning model to distinguish ATC from DTC in TCGA based on 5 key genes. **(G)** The standardized net benefit and high-risk threshold were calculated between MTC and DTC. **(H)** Machine learning model to distinguish MTC from DTC in GEO datasets based on 5 key genes. **(I)** Machine learning model to distinguish MTC from DTC in TCGA based on 5 key genes. **(J)** The standardized net benefit and high-risk threshold were calculated between FTC and DTC. **(K)** Machine learning model to distinguish FTC from DTC in GEO datasets based on 5 key genes. **(L)** Machine learning model to distinguish FTC from DTC in TCGA based on 5 key genes. RF, Random Forest; SVM, Support Vector Machine; XGB, eXtreme Gradient Boosting; GLM, Generalized Linear Model; GBM, Gradient Boosting Machine; KKN, Kernel k-Nearest Neighbors; NNET, Neural Network; LASSO, Least Absolute Shrinkage and Selection Operator.

BCL2, BHLHE40, MICAL2, TGM2, and TPO were further employed to make a model to differentially diagnose TCA from TNC (**Figures 2A–C**), ATC from DTC (**Figures 2D–F**), MTC from DTC (**Figures 2G–I**), and FTC from DTC (**Figures 2J–L**). Different machine learning algorithms, for instance, Random Forest (RF), Support Vector Machine (SVM), eXtreme Gradient Boosting (XGB), Generalized Linear Model (GLM), Gradient Boosting Machine (GBM), Kernel k-Nearest Neighbors (KKNN), Neural Network (NNET), Least Absolute Shrinkage and Selection Operator (LASSO) were used to select the best model. With six of eight AUC values more than 0.9 (**Figures 2B, E, H, K, I, L**), the model based on RF was validated as the best one in both TCGA and GEO databases.

### 3.3 The immune cell infiltration varied significantly among the ATC subgroups C1, C2, and C3

**Figure 3:**
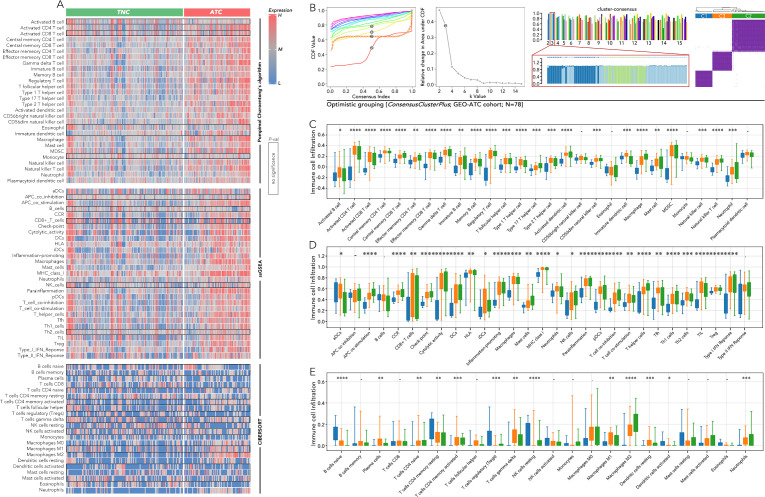
Immune cell infiltration analysis among anaplastic thyroid cancer (ATC) subgroups based on 5 key genes. **(A)** Immune cell infiltration was calculated by Pompimol Charoentong’s algorithm, ssGSEA, and CIBERSORT compared in TNC and ATC. **(B)** Consensus clustering based on the previous 5 genes divided the ATC into 3 three subgroups (C1, C2, C3). **(C)** Immune cell infiltration calculated by Pompimol Charoentong’s algorithm was compared among C1, C2, and C3. **(D)** Immune cell infiltration calculated by ssGSEA was compared among C1, C2, and C3. **(E)** Immune cell infiltration calculated by CIBERSORT was compared among C1, C2, and C3. SsGSEA (single sample GSEA) P value < 0.05 (*), P value < 0.01 (**), P value < 0.001 (***), P value < 0.0001 (****).

The immune cell infiltration between TNC and ATC, calculated by three methods (Pompimol Charoentong’s algorithm, ssGSEA and CIBERSORT), was presented in a heatmap. A majority of immune cell types could infiltrate the tumor microenvironment in ATC (**Figure 3A**). Consensus clustering based on the previous 5 genes was harnessed to categorize ATC into three distinct groups: C1, C2, and C3 (**Figure 3B**). The immune cell infiltration in the three ATC subgroups was further calculated using three well-recognized methods. Approximately 85% of the immune cell types, including activated CD4 T cells, central memory CD8 T cells, effector memory CD8 T cells, dendritic cells, and macrophages, exhibited differential infiltration among C1-C3. Significant differences in activated CD8 T cell infiltration were observed in the results from the first two methods (**Figures 3C–E**).

### 3.4 The predicted immunotherapy response differed among the ATC subgroups C1, C2, and C3

**Figure 4:**
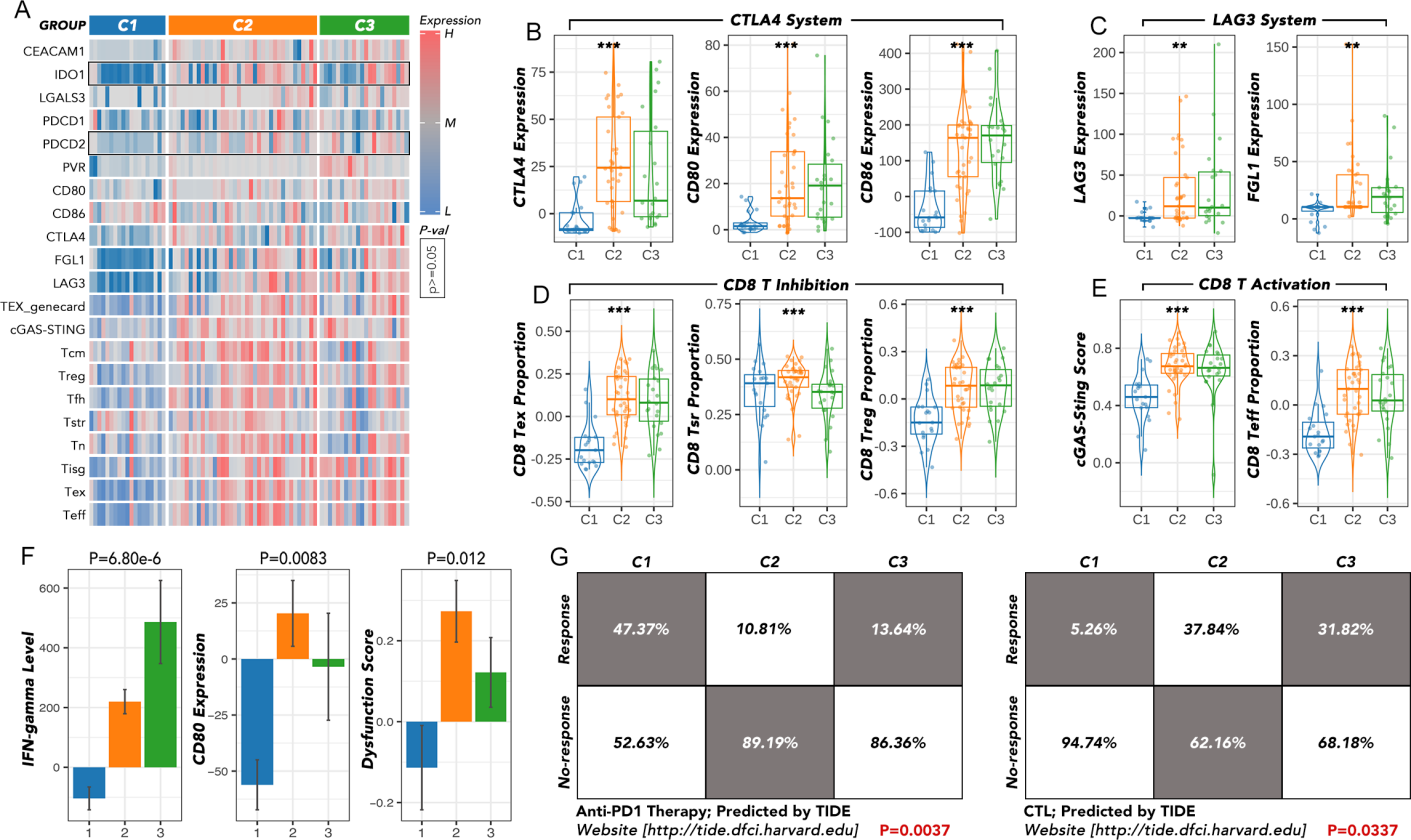
The immunotherapy response analysis among the ATC subgroups. **(A)** The expression of key immune checkpoints was compared among C1, C2, and C3. **(B)** The expression of the CTLA4 system was compared among C1, C2 and C3. **(C)** The expression of the LAG3 system was compared among C1, C2 and C3. **(D)** The expression of the CD8 T inhibition family was compared among C1, C2, and C3. **(E)** The expression of the CD8 T activation family was compared among C1, C2 and C3. **(F)** The IFN-gamma level, CD80 expression, and dysfunction score were compared among C1, C2, and C3. **(G)** The response rates to anti-PD1 or CTL therapy were predicted by TIDE. TEX, T cell exhaustion; T cm, central memory T cell; Treg, regulatory T cells; T fh, T follicular helper cells; T sr, T cells in a stress response state; T n, naïve T cell; T isg, IFN stimulated regulator T cell; T eff, effective T cell; TIDE, Tumor Immune Dysfunction and Exclusion; CTL, cytotoxic T lymphocyte. **p<0.01, ***p<0.001.

The expression of key immune checkpoints was compared among the three groups (**Figure 4A**). CTLA4, CD80, and CD86 (CTLA4 system) were most highly expressed in the C2 group and least expressed in the C1 group (**Figure 4B**). LAG3 and FGL1 exhibited a similar expression pattern to the CTLA4 system (**Figure 4C**). Inhibitory markers of CD8 T cells, including T cell exhaustion (TEX) and regulatory T cells (Treg), were more highly expressed in the C2 and C3 groups, while markers of T cells in a stress response state (T sr) were relatively higher in the C1 and C2 groups (**Figure 4D**). Activating markers of CD8 T cells, such as the cGAS-STING score and CD8 effector T cells (Teff), showed a similar expression pattern to TEX and Treg (**Figure 4E**). Other immune markers, including IFN-gamma, CD80, and dysfunction score, were also enriched in the C2 and C3 groups (**Figure 4F**). According to the Tumor Immune Dysfunction and Exclusion (TIDE) analysis, patients in the C1 group may benefit from anti-PD1 therapy, whereas those in the C2 and C3 groups may be more suitable for cytotoxic T lymphocyte (CTL) therapy (**Figure 4G**).

### 3.5 12 innovative genes identified by WGCNA effectively distinguished C1-C3 groups using machine learning

**Figure 5:**
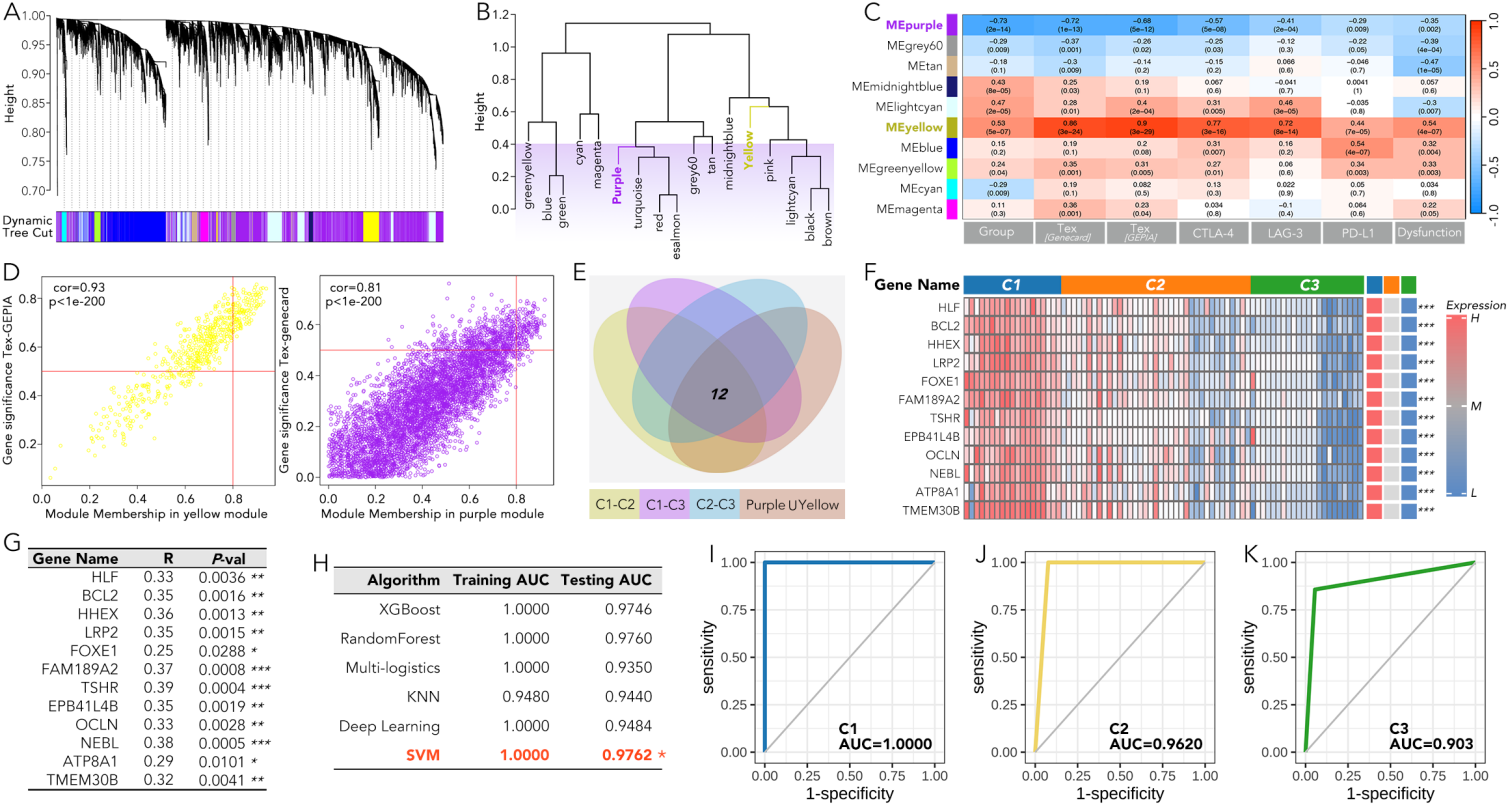
A machine-learning grouping model for anaplastic thyroid cancer (ATC) based on the 12 genes most related to the ATC grouping and immune pathways in WGCNA. **(A-D)** The WGCNA results were presented based on the grouping, TEX, CTLA-4, LAG-3, PD-L1, and immune dysfunction score. **(E)** 12 genes in the purple or yellow module were expressed differently (P value > 0.05 and a fold change (|FC|) > 2) between any pair in the C1, C2, and C3 group. **(F)** The expression of the 12 genes in C1-C3 groups was shown by heatmap. **(G)** The correlation coefficients were calculated between the individual level of the 12 genes and the TEX. **(H-K)** A machine learning model based on SVM was used to distinguish C1, C2, and C3 on these 12 genes. T cell exhaustion (TEX). *P<0.05, **p<0.01, ***p<0.001.

Based on the weighted gene co-expression network analysis (WGCNA), the purple gene module was most negatively correlated with the grouping, TEX, CTLA-4, LAG-3, PD-L1, and immune dysfunction score, while the yellow module was most positively correlated with these markers (**Figures 5A–D**). Further analysis, overlapping the differentially expressed genes between each pair of C1-C3 groups (P value > 0.05 and a fold change (|FC|) > 2) with the combined gene set of both the yellow and purple modules, identified 12 genes (HLF, BCL2, HHEX, LRP2, FOXE1, FAM189A2, TSHR, EPB41L4B, OCLN, NEBL, ATP8A1, and TMEM30B) that may play an important role in the immune therapy of ATC (**Figure 5E**). The expression of these 12 genes showed consistent patterns within the C1-C3 groups (high in C1, intermediate in C2, and low in C3) (**Figure 5F**). Moreover, the individual expression of these 12 genes was positively related to TEX (**Figure 5G**). The subgrouping model by SVM, based on these 12 genes, effectively distinguished the C1-C3 groups with an AUC over 0.9 (**Figures 5H–K**).

### 3.6 Grouping based on the 12 genes across 9 melanoma cohorts receiving anti-PD1 therapy revealed significantly different response rates

**Figure 6:**
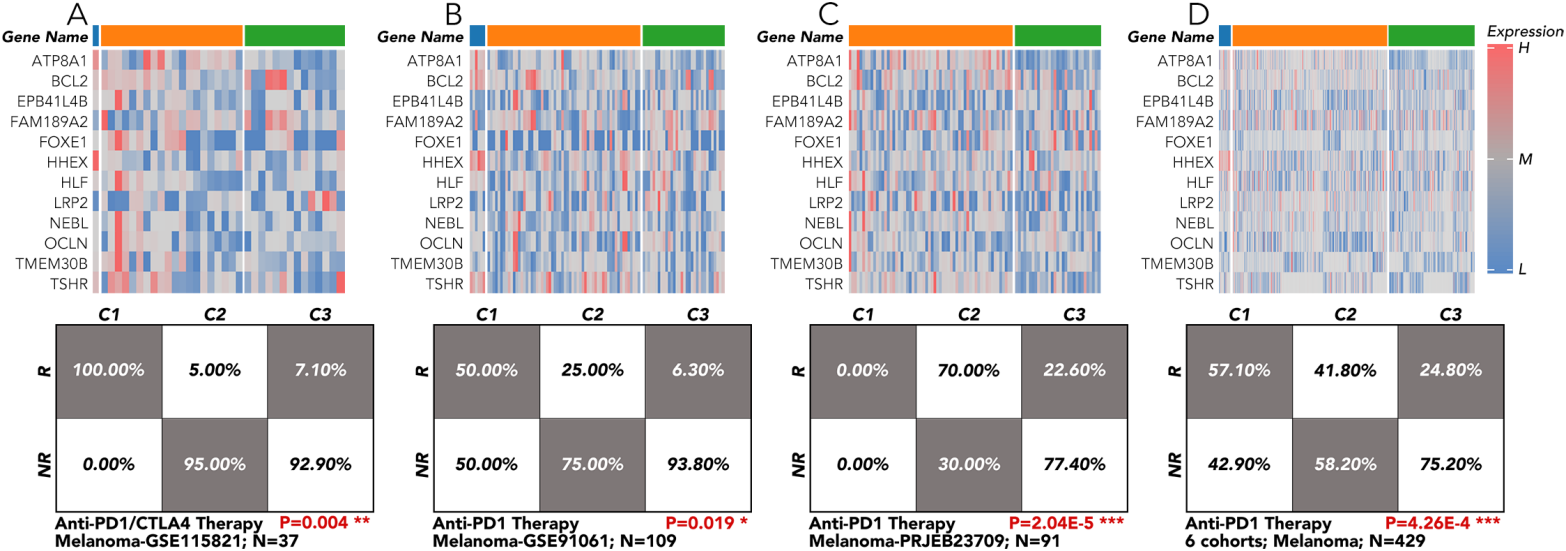
Immunotherapy response validation by the 12-gene grouping model in TCGA-THCA and clinical cohort of melanoma. **(A)** The 12-gene signature was applied in the melanoma cohort from GSE115821 to test the response rate to anti-PD1/CTLA4 therapy. **(B)** The 12-gene signature was applied in the melanoma cohort from GSE91061 to test the response rate to anti-PD1 therapy. **(C)** The 12-gene signature was applied in the melanoma cohort from PRJEB23709 to test the response rate to anti-PD1 therapy. **(D)** The 12-gene signature was applied in the 6 melanoma cohorts to test the response rate to anti-PD1 therapy. All the data of the melanoma cohorts were downloaded from the website (http://tide.dfci.harvard.edu/login/). *P<0.05, **p<0.01, ***p<0.001.

**Figure 7:**
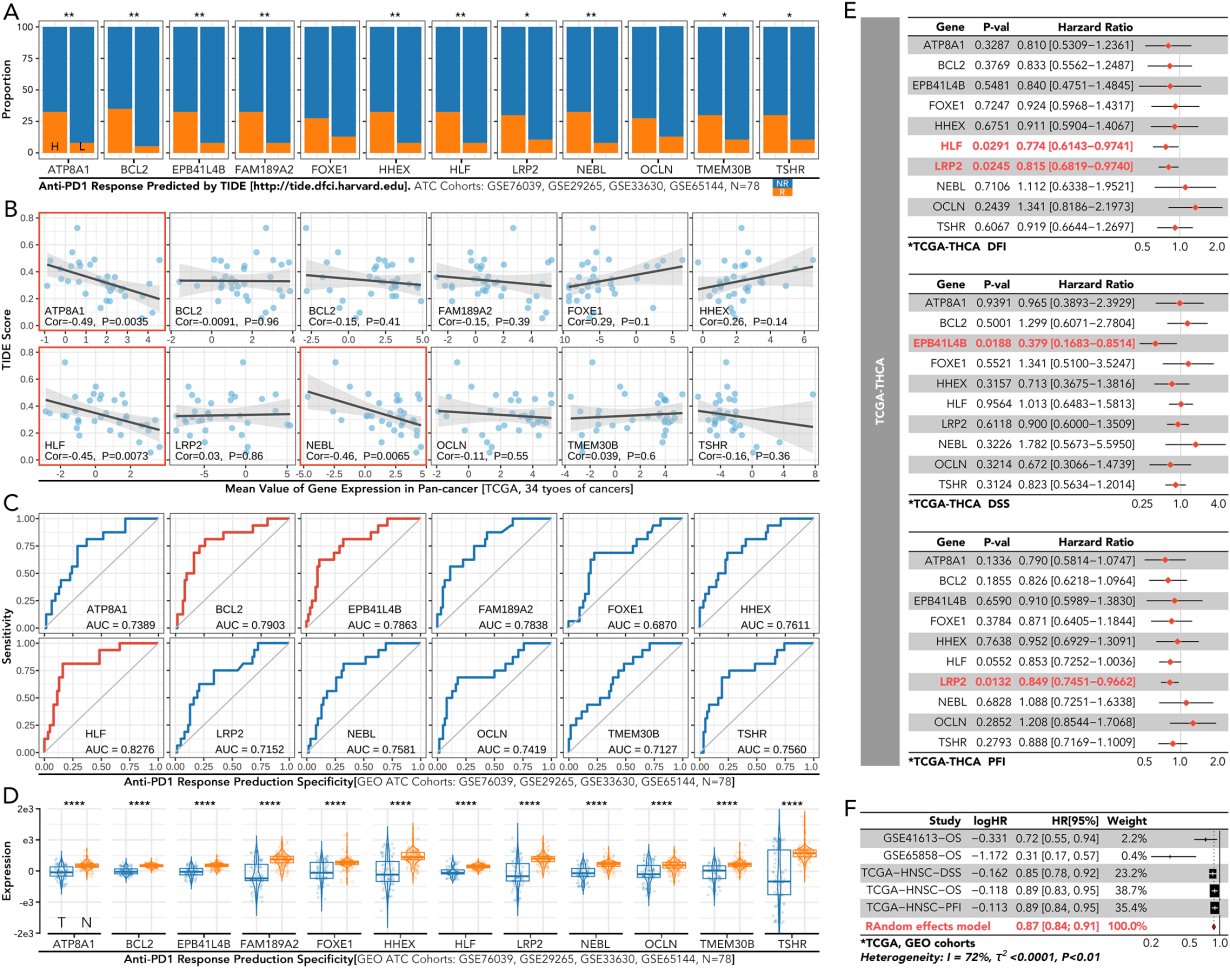
Immune response and prognosis analysis of the individual gene from the 12-gene signature in thyroid cancer. **(A)** Predicted response rates to anti-PD1 therapy, calculated by TIDE, were compared between high-expression (left) and low-expression (right) groups for each gene in the 12-gene signature. **(B)** The correlation between the TIDE score and the expression of the individual gene in the 12-gene signature. **(C)** ROC curves for predicting anti-PD1 therapy response based on each gene. **(D)** Comparison of each gene’s expression between cancerous **(CA)** and non-cancerous (NC) thyroid tissues in the GEO dataset. **(E)** Cox regression of each gene for DFI/DSS/PFI in TCGA-THCA **(F)** Cox regression analysis of each gene for OS in GSE41613, GSE65858, and TCGA-HNSC, and for DSS and PFI in TCGA-HNSC. NR, No response; ROC, receiver operating characteristic; CA, cancerous; NC, non-cancerous; DFI, disease-free interval; DSS, disease-specific survival; PFI, progression-free interval; TCGA-THCA, the cancer genome atlas-thyroid cancer; OS, overall survival; TCGA-HNSC, the cancer genome atlas-head and neck squamous cell carcinoma. *P<0.05, **p<0.01.

The grouping model based on the 12 innovative genes was validated in available clinical trial cohorts. Except in the melanoma-PRJEB23709 cohort, the actual response rates to anti-PD1 therapy in the C2 and C3 groups were much lower than in the C1 group, consistent with our results (**Figures 4G**; **6A–D**). The correlation of individual gene expression with the predicted response rate to anti-PD1 therapy, as determined by TIDE, was further analyzed (**Figure 7A**). HLF, ATP8A1, and NEBL stood out due to their correlation coefficients over 0.4 and AUC values over 0.75 (**Figures 7B, C**). All components of the 12-gene signature were down-regulated in the TCGA thyroid cancer samples compared to the non-cancer samples (**Figure 7D**). Of the three genes, only HLF was of prognostic significance in the disease-free interval (DFI) of TCGA thyroid carcinoma (THCA) patients (**Figure 7E**). More importantly, its pro-survival role in prognosis was further corroborated in overall survival (OS) from three head and neck squamous cell carcinoma (HNSC) cohorts (GSE41613, GSE65858, TCGA). Its role was also validated in disease-specific survival (DSS) and progression-free interval (PFI) in the TCGA-HNSC cohort (**Figure 7F**).

### 3.7 HLF was a tumor suppressor in pan-cancer and could promote T-cell infiltration in ATC

**Figure 8:**
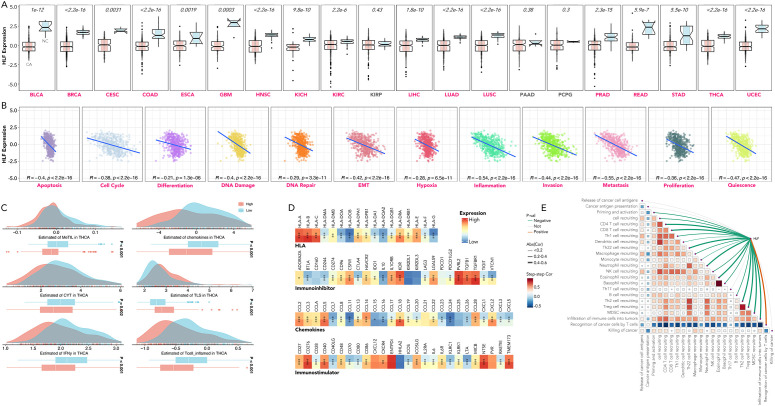
Dissection of HLF’s relation with cancer hallmarks in pan-cancer. **(A)** HLF expression in CA versus NC tissues across BLCA, BRCA, CESC, COAD, ESCA, GBM, HNSC, KICH, KIRC, KIRP, LIHC, LUAD, LUSC, PAAD, PCPG, PRAD, READ, STAD, THCA, and UCEC. **(B)** Correlation between HLF expression and apoptosis, cell cycle regulation, differentiation, DNA damage and repair, EMT, hypoxia, inflammation, invasion, metastasis, proliferation, and quiescence in TCGA-THCA. **(C)** Comparison of MeTIL, chemokines, CYT, TLS, IFN-gamma, and inflamed T cells between HLF low-expression and high-expression ATC. **(D)** Comparison of HLA, immunoinhibitor family, chemokines, and immunostimulator family between HLF low-expression and high-expression ATC. **(E)** Correlation between HLF expression and recruitment of various types of immune cells in TCGA-THCA. CA, Cancerous; NC, non-cancerous; BLCA, bladder urothelial carcinoma; BRCA, breast invasive carcinoma; CESC, cervical squamous cell carcinoma, endocervical adenocarcinoma; COAD, colon adenocarcinoma; ESCA, esophageal carcinoma; GBM, glioblastoma multiforme; HNSC, head and neck squamous cell carcinoma; KICH, kidney chromophobe; KIRC, kidney renal clear cell carcinoma; KIRP, kidney renal papillary cell carcinoma; LIHC, liver hepatocellular carcinoma; LUAD, lung adenocarcinoma; LUSC, lung squamous cell carcinoma; PAAD, pancreatic adenocarcinoma; PCPG, pheochromocytoma and paraganglioma; PRAD, prostate adenocarcinoma; READ, rectum adenocarcinoma; STAD, stomach adenocarcinoma; THCA, thyroid carcinoma; UCEC, uterine corpus endometrial carcinoma; EMT, epithelial-mesenchymal transition; CYT, cytolytic activity; MeTIL, methylation status of genes in tumor-infiltrating lymphocytes; TLS, tertiary lymphoid structures; HLA, human leukocyte antigen. *P<0.05, **p<0.01, ***p<0.001.

Comparative analysis between cancerous (CA) and non-cancerous (NC) tissues revealed that HLF expression was generally lower in CA, observed in 17 out of 20 types (**Figure 8A**). The expression of HLF was negatively related to markers of apoptosis, cell cycle regulation, differentiation, DNA damage and repair, epithelial-mesenchymal transition (EMT), hypoxia, inflammation, invasion, metastasis, proliferation, and quiescence in TCGA-THCA patients (**Figure 8B**). Significant differences were also observed in the methylation status of genes in tumor-infiltrating lymphocytes (MeTIL), cytolytic activity (CYT), tertiary lymphoid structures (TLS), human leukocyte antigen (HLA), immunoinhibitor family, immunostimulator family, immune cell recruitment, and other immune markers between HLF high-expression and low-expression groups (**Figures 8C–E**).

**Figure 9:**
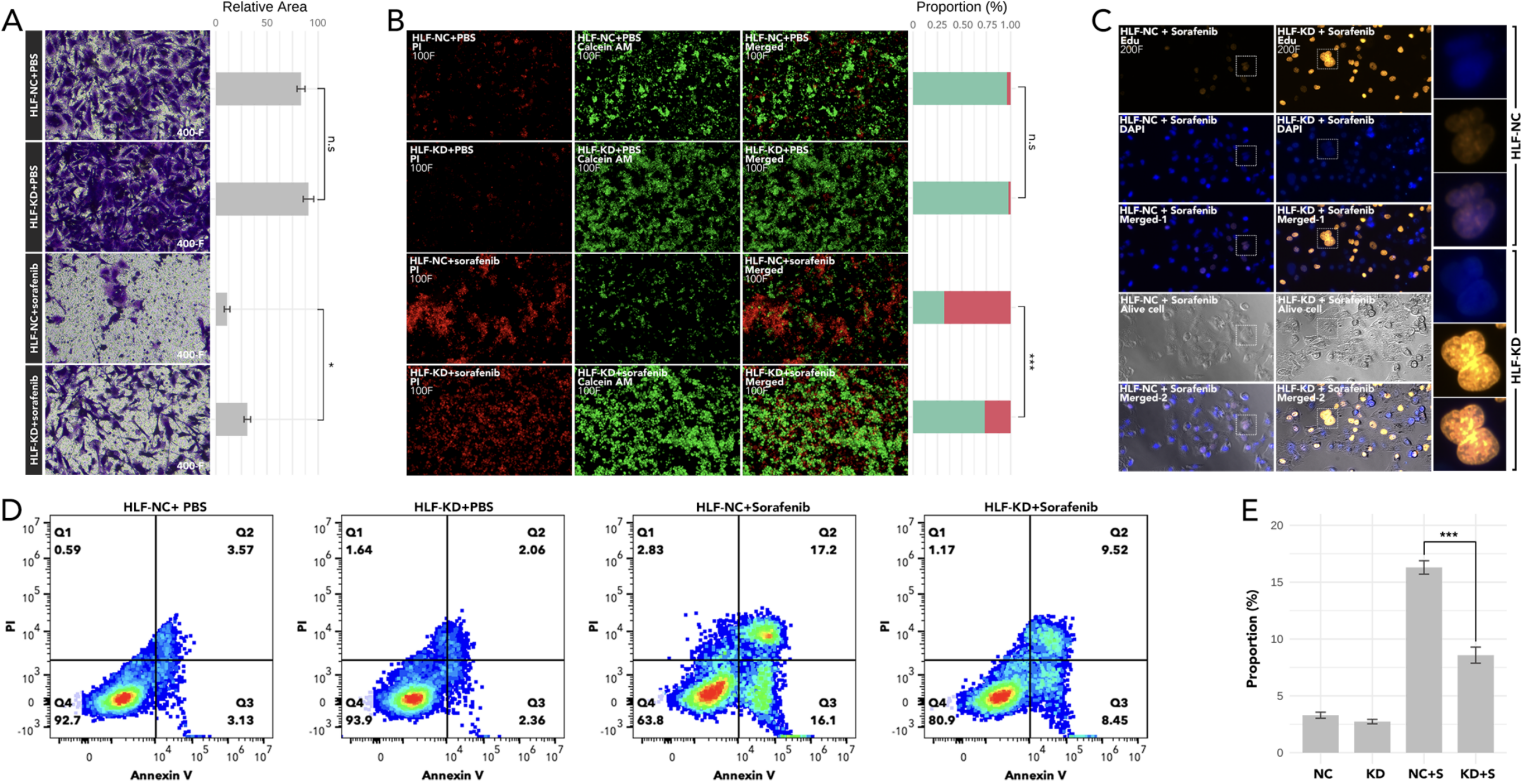
Analysis of cell migration, viability, proliferation, and apoptosis following HLF knockdown in ATC cell line CAL62. **(A)** Cell migration assay comparing CAL62 vs. HLF-KD CAL62 and CAL62 treated with sorafenib (4μM) vs. HLF-KD CAL62 treated with sorafenib (4μM). **(B)** Dead (PI)/live staining (calcein AM) detection comparing CAL62 vs. HLF-KD CAL62 and CAL62 treated with sorafenib (4μM) vs. HLF-KD CAL62 treated with sorafenib (4μM). **(C)** Proliferation assay (Edu staining) comparing CAL62 treated with sorafenib (4μM) and HLF-KD CAL62 treated with sorafenib (4μM). **(D)** Apoptosis detection in CAL62 vs. HLF-KD CAL62, and CAL62 treated with sorafenib (4μM) vs. HLF-KD CAL62 treated with sorafenib (4μM). **(E)** Comparison of early apoptosis as shown in **(D)**. HLF-KD indicates HLF knockdown by siRNA. *P<0.05, ***p<0.001.

**Figure 10:**
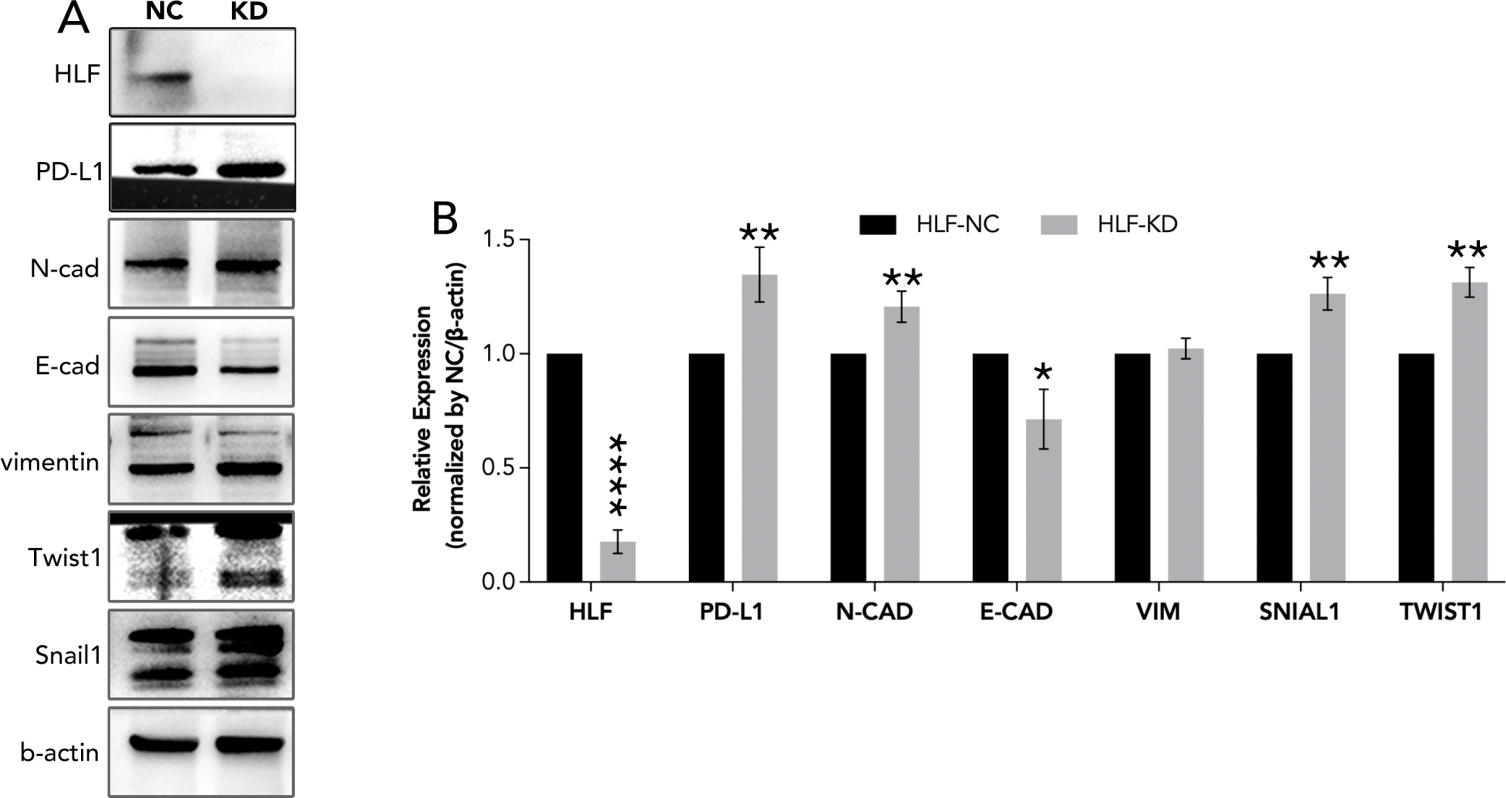
EMT pathway detection following HLF knockdown in ATC cell line CAL62. **(A)** EMT pathway detection after HLF knockdown in CAL62. **(B)** Quantitative analysis of protein expression as shown in **(A)**. EMT, Epithelial-mesenchymal transition. *P<0.05, **p<0.01.

**Figure 11:**
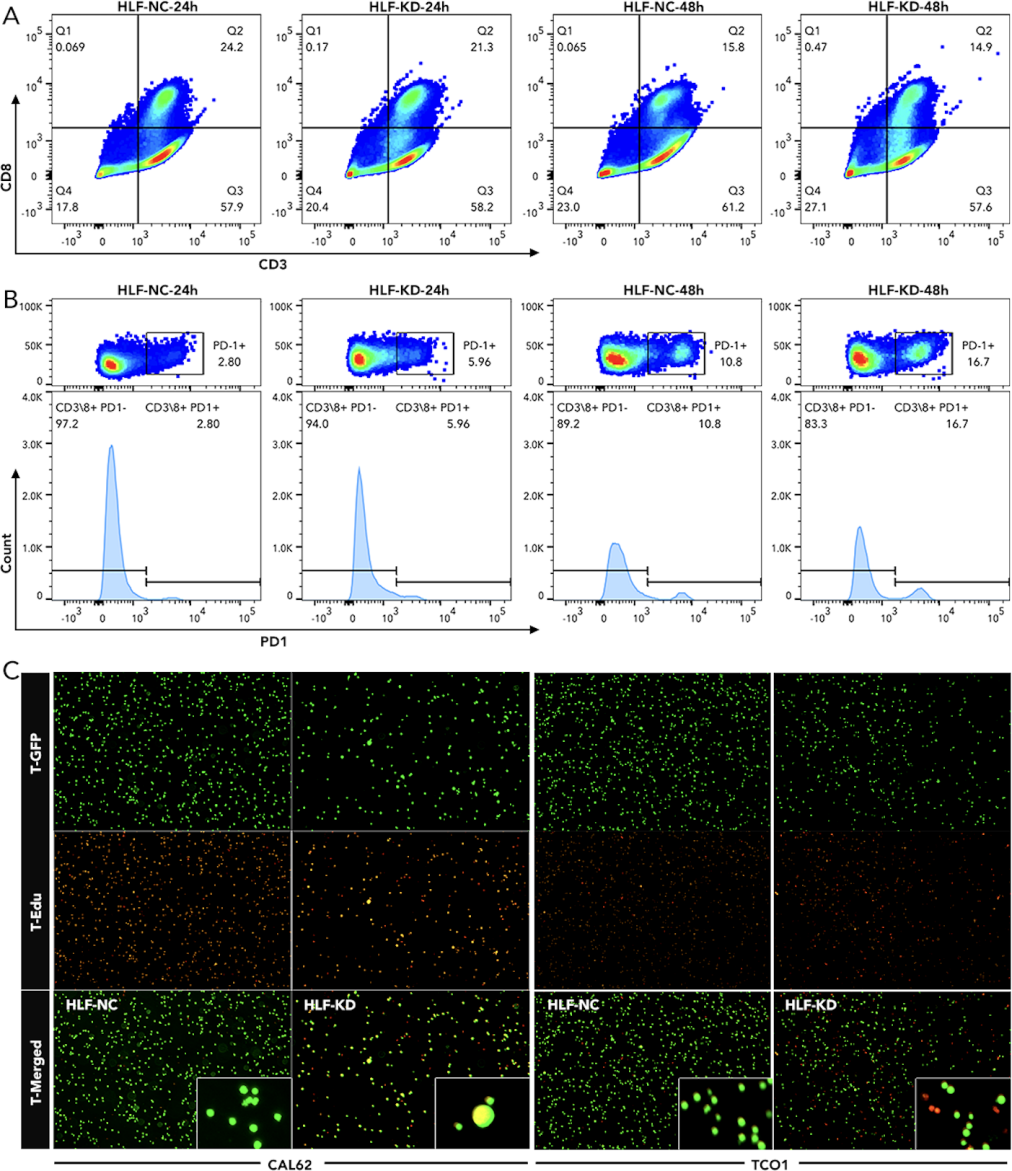
Detection of T-cell exhaustion and chemotaxis following HLF knockdown in ATC cell lines **(A)** Detection of CD8 T cells in PBMC co-cultured with CAL62 for 24 hours or 48 hours. **(B)** Detection of PD1 in CD8 T cells as shown in Q2 of **(A)**. **(C)** T-cell (Jurkat cells) chemotaxis assay following HLF knockdown in ATC cell line CAL62 and TCO1. EMT, Epithelial-mesenchymal transition; PBMC, peripheral blood mononuclear cell.

**Figure 12:**
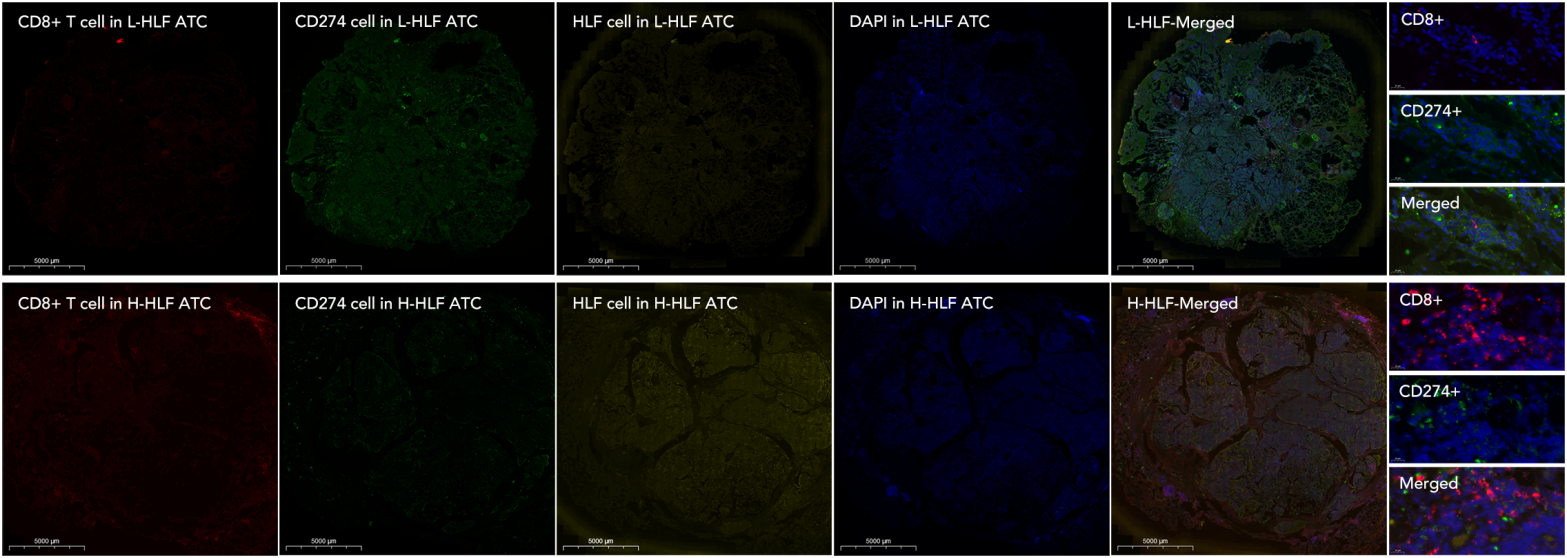
T cell infiltration detection in HLF low-expression and high-expression ATC samples. The immunofluorescence intensity of CD8, CD274/PD-L1, HLF, and DAPI was compared between L-HLF and H-HLF ATC. L-HLF, HLF low-expression; H-HLF, HLF high expression.

Knockdown of HLF in ATC cell line CAL62 could greatly increase the migration of this cell line. A similar trend was also observed in the treatment of sorafenib in the lower layer (4μM) (**Figure 9A**). Dead/live cell staining using PI and calcein AM, proliferation staining by EDU, along with apoptosis detection demonstrated that HLF-knockdown CAL62 cells exhibited a lower proportion of dead cells, a higher proportion of live cells, an increased proliferation rate, and enhanced resistance to sorafenib (**Figures 9B–E**). The EMT pathway was upregulated in HLF-knockdown CAL62 cells (**Figures 10A, B**). Co-culturing CAL62 (in the lower layer) with peripheral blood mononuclear cells (PBMCs) (in the upper layer) using transwells indicated that HLF knockdown in CAL62 induced more PD1+ CD8 T cells (**Figures 11A, B**). Further co-culturing of CAL62/TCO1 ATC cells (in the lower layer) with Jurkat T cells (in the upper layer) showed that T cell recruitment decreased following HLF knockdown in the ATC cell line (**Figure 11C**). Finally, in 22 ATC clinical samples from our hospital, CD8 T cells and PD-L1/CD274 were detected, revealing an increase in PD-L1 and a decrease in CD8 T cells in the HLF low-expression samples (**Figure 12**).

## 4 Discussion

To uncover the most distinct genes in different thyroid cancer subtypes, we screened BCL2, BHLHE40, MICAL2, TGM2, and TPO by comparing each pair of existing subtypes (**Figure 1**). BCL-2 (B-cell lymphoma 2) is a protein that plays a crucial role in regulating apoptosis (14, 15). Some researchers have found that BCL-2 might help thyroid cancer evade apoptosis, making it a suitable target for therapy (16–18). In our study, BCL-2 levels were relatively lower in ATC but higher in TNC, suggesting it may not be a promising target for ATC. BHLHE40 has been reported to be involved in the aggressiveness of various cancers, including colorectal, pancreatic, and endometrial cancers (19–22). One study focusing on ATC revealed that the lncRNA/H19-miR-454-3p/BHLHE40 axis could potentiate the progression of ATC (23). Consistent with this, BHLHE40 expression was significantly higher in TCA compared to TNC, and its expression in ATC was the second highest among all subtypes. MICAL2 is known as an oncogene in many cancers, such as pancreatic, ovarian, and gastric cancers (24–26), while there is no related research on thyroid cancer. Our results indicated that MICAL2 may also play a role in ATC. A similar phenomenon was observed with TGM2, which has been proven to be an oncogene with little research on ATC (27, 28). TPO has been reported to predict higher metastasis and recurrence in PTC (29). It was nearly unexpressed in ATC in our results. Our findings revealed that BCL2, BHLHE40, MICAL2, TGM2, and TPO were expressed differently in ATC, FTC, MTC, PTC, and TNC. This inspired us to construct a signature based on these genes to help differentiate the subtypes. The model using the RF machine learning method worked precisely in differentiating TCA from TNC, ATC from DTC, MTC from DTC, and FTC from DTC (**Figure 2**). However, the mechanisms by which these genes differentiate the subtypes and their roles in ATC, especially MICAL2, still require further research.

Since the five genes were so distinct, we continued to divide the most aggressive type, ATC, into more subtypes by consensus clustering, which may provide clues for specific therapy selection. The great variation of immune cell infiltration in the three subgroups indicated that immune therapy selection may differ among them. Further prediction by TIDE implied that the C1 group could more likely benefit from anti-PD1 therapy, while the C2 and C3 groups may be more suitable for CTL therapy (**Figures 3**, **4**). Until now, immunotherapy (anti-PD-1 and anti-PD-L1) has shown the most promising 1-year survival rate of approximately 40% for ATC patients without BRAF and MEK gene mutations (7). There is no CTL therapy for ATC patients in clinical trials. Our findings imply that grouping ATC patients may maximize the efficacy of immune therapy. More real-world clinical data are still needed to test this hypothesis.

To further characterize the gene expression patterns among C1, C2, and C3 groups in ATC, WGCNA was used to identify the most associated gene modules. Twelve genes (HLF, BCL2, HHEX, LRP2, FOXE1, FAM189A2, TSHR, EPB41L4B, OCLN, NEBL, ATP8A1, and TMEM30B) were selected from the most related modules due to their distinct expression among the three subgroups. A more precise model based on these 12 genes was created to replace the previous 5-gene signature. More importantly, when the new model was retrospectively applied to the available real-world melanoma clinical cohort, it validated that the C1 group was more suitable for anti-PD1 therapy (**Figures 5**, **6**). However, we must acknowledge that one result from the nine cohorts did not comply with our conclusion. In the future, we hope to test the model using data from ATC patients receiving anti-PD1 therapy or CTL therapy.

HLF was chosen from the 12 genes for final experimental validation because it performed well in both immune therapy response prediction and prognosis prediction in external cohorts (**Figure 7**). As a transcriptional activator, HLF has been validated as a tumor suppressor in triple-negative breast cancer and ovarian cancer (30, 31). However, one study indicated that it could promote the development of hepatocellular carcinoma and resistance to sorafenib (32). No research on HLF has been found in ATC. Our study indicated that the knockdown of HLF could promote the migration, proliferation, survival and sorafenib resistance of ATC cell lines (**Figure 9**). The up-regulation of EMT pathway in the HLF-knockdown group may explain its role as a tumor suppressor in ATC (**Figure 10**). The increased T-cell exhaustion, indicated by up-regulated PD-1 after 48 hours, and the dampened T-cell recruitment were also observed following the decrease in HLF expression in ATC cell lines (**Figures 11**, **12**). More interestingly, PD-L1/CD274 was also up-regulated in the HLF-low-expression group (**Figures 10**, **12**). These results suggest that HLF may be necessary for immune cells to function normally in the ATC tumor environment. Although further research is needed to explore HLF’s role in the ATC microenvironment, to our knowledge, this study is the first to investigate HLF in ATC. We will continue to explore this in our future studies.

## 5 Conclusion

In summary, our study identified five genes with distinct expression patterns across all subtypes of thyroid cancer. A signature based on these five genes can precisely distinguish between the subtypes. Additionally, our group developed a 12-gene signature in ATC that can predict the response to anti-PD1 therapy to some extent. The tumor suppressor role of HLF was validated in ATC cell lines through *in vitro* experiments.

## Data Availability

The original contributions presented in the study are included in the article/supplementary materials, further inquiries can be directed to the corresponding author/s.
